# Long noncoding RNAs as auxiliary biomarkers for gastric cancer screening: A pooled analysis of individual studies

**DOI:** 10.18632/oncotarget.8268

**Published:** 2016-03-22

**Authors:** Zhaolei Cui, Yan Chen, Zhenzhou Xiao, Minhua Hu, Yingying Lin, Yansong Chen, Yuhong Zheng

**Affiliations:** ^1^ Department of Clinical Laboratory, Fujian Provincial Key Laboratory of Tumor Biotherapy, Fujian Provincial Cancer Hospital, Teaching Hospital of Fujian Medical University, Fuzhou, Fujian, China

**Keywords:** lncRNA, gastric cancer, biomarker, diagnostic accuracy, meta-analysis

## Abstract

**Background:**

Long non-coding RNAs (lncRNAs) are highlighted as novel cancer biomarkers with great promise. Herein, we focused on summarizing the overall diagnostic performance of lncRNAs for gastric cancer (GC).

**Methods:**

Publications fulfilling the search criteria were selected from the online databases. Study quality was assessed according to the Quality Assessment for Studies of Diagnostic Accuracy (QUADAS) checklist. The summary receiver operator characteristic (SROC) curve was plotted using a bivariate meta-analysis model. Statistical analysis was performed based on the platforms of STATA 12.0 and Meta-Disc 1.4 software.

**Results:**

Fifteen studies with 1252 patients and 1283 matched controls were included. The pooled sensitivity and specificity for lncRNA expression profile in differentiating GC patients from cancer-free individuals were 0.68 (95%CI: 0.61-0.74) and 0.79 (95%CI: 0.72-0.84), respectively, corresponding to an area under curve (AUC) of 0.80. Moreover, the stratified analyses demonstrated that plasma-based lncRNA profiling harbored higher accuracy than that tissue-based assay (specificity: 0.80 versus 0.75; AUC: 0.84 versus 0.77).

**Conclusions:**

LncRNA profiling hallmarks a moderate diagnostic value in the management of GC and that lncRNA expression patterns may potentially be utilized as auxiliary biomarkers in confirming GC.

## INTRODUCTION

Gastric cancer (GC) remains a common occurring malignancy among human cancers, representing the second leading cause of cancer deaths worldwide [1, 2]. Despite recent advances in medical technology for GC, the overall 5-year survival rate is still less than 30% [3]. Due to a lack of typical early symptoms, most GC patients are diagnosed at an advanced stage with high lymph node metastasis. Hence, early diagnosis is an important way to improve the overall survival rate of GC. Endoscopy is currently the most reliable diagnostic tool for early GC detection, but this method has suffered from lots of disadvantages as invasive, high cost and low efficient, etc [4]. The examination of blood tumor biomarkers as pepsinogen (PG), MG-7, carbohydrate antigen 19-9 (CA19-9), carbohydrate antigen (CA72-4) and carcinoembryonic antigen (CEA) are also available [4–6]. However, the accuracies of these markers are not yet satisfactory. In this respect, the identification of tumor markers for early GC diagnosis is highly needed in clinic.

Long noncoding RNAs (lncRNAs) are a class of newfound RNAs that greater than 200 bp but with no protein-coding capability [7]. In recent years, lncRNAs are rapidly gaining prominence due to the discovery of their crucial and functional importance in cancer occurrence and progression. Many human cancers include GC are frequently associated with altered lncRNA signature [8–10]. In addition to the role in tumor occurrence and progression, the diagnostic value of lncRNAs for GC was highlighted as well [11–26]. However, views on the diagnostic accuracy of lncRNAs were inconsistent, even conflictive among literature. Consequently, we conducted this meta-analysis and aimed to get an overall understanding of lncRNAs in diagnosing GC.

## RESULTS

### Literature search

**Figure 1:**
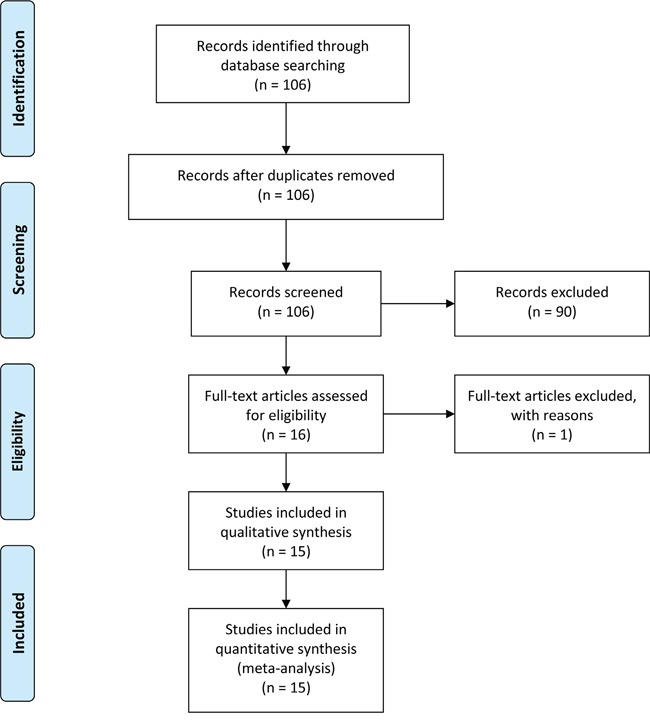
Flow diagram of study selection process

Our initial literature search identified a total of 106 relevant publications concerning the current topic. The titles and abstracts from each article were carefully reviewed, and 90 of them were further excluded due to the status of review articles, letters, basic research, and so forth. The retrieved 16 studies received full test review, and 1 study was finally discarded due to the lack of sufficient data [12]. Hence, only 15 articles were screened out in this meta-analysis [11, 13–26]. The process of study selection was presented in Figure 1.

### Study characteristics and quality assessments

The 15 studies involved 1252 patients and 1283 matched controls, and the patient size in each study varied from 17 to 138 and all the GC patients had a definite diagnosis through the histopathological method. The quantitative reverse transcription PCR (qRT-PCR) method was used in evaluating lncRNA levels, and the reference gene contained GAPDH [11, 13, 15, 16, 18, 19, 20, 22-24] and β-actin [14, 17, 21, 25, 26]. Specimen type included plasma [11, 13, 14], serum [21], gastric juice [23] and tissue [15-20, 22, 24-26]. All studies were conducted in Chinese population. The main features of enrolled studies were displayed in Table 1.

**Table 1:** Main feature of the included studies

Author	Year	Population	Patients (Controls)	Control sources	Sample type	Cut-off value	Method	LncRNA signatures	Expression status	QUADAS scores
Chen et al. [22]	2014	Chinese	94 (94)	ANT	Tissue	Unclear	qRT-PCR	AC138128.1	Decreased	11
Chen et al. [18]	2015	Chinese	83 (83)	ANT	Tissue	9.56	qRT-PCR	HIF1A-AS2	Increased	12
Dong et al. [21]	2015	Chinese	90 (86)	Nontumorous mucosa	Serum	Unclear	qRT-PCR	CUDR, LSINCT-5 and PTENP1	Decreased	11
Li et al. [13]	2014	Chinese	79 (81)	Healthy blood	Plasma	Unclear	qRT-PCR	LINC00152	Increased	10
Lin et al. [24]	2014	Chinese	75 (75)	ANT	Tissue	<11.0	qRT-PCR	ABHD11-AS1	Increased	12
Liu et al. [14]	2014	Chinese	83 (80)	Healthy blood	Plasma	15.43	qRT-PCR	FER1L4	Decreased	12
Liu et al. [15]	2014	Chinese	138 (138)	ANT	Tissue	4.97	qRT-PCR	NcRuPAR	Decreased	12
Mei et al. [26]	2013	Chinese	96 (96)	ANT	Tissue	2.31	qRT-PCR	SUMO1P3	Increased	12
Pang et al. [16]	2014	Chinese	17 (16)	Normal tissue	Tissue	4.385	qRT-PCR	LINC00152	Increased	12
Sun et al. [17]	2013	Chinese	78 (78)	ANT	Tissue	13.955	qRT-PCR	AC096655.1-002	Decreased	12
Sun et al. [20]	2015	Chinese	96 (96)	ANT	Tissue	6.445	qRT-PCR	RP11-119F7.4	Decreased	11
Shao et al. [23]	2014	Chinese	83 (120)	ANT	Gastric juice	0.88	qRT-PCR	AA174084	Decreased	12
Zhao et al. [25]	2014	Chinese	58 (58)	ANT	Tissue	10.88	qRT-PCR	HULC	Increased	12
Zheng et al. [19]	2015	Chinese	112 (112)	ANT	Tissue	13.74	qRT-PCR	UCA1	Increased	12
Zhou et al. [11]	2015	Chinese	70 (70)	Healthy blood	Plasma	Unclear	qRT-PCR	H19	Increased

Abbreviations: ANT: adjacent non-tumor tissues; QUADAS: quality assessment for studies of diagnostic accuracy; qRT-PCR: quantitative reverse transcription PCR.

**Figure 2:**
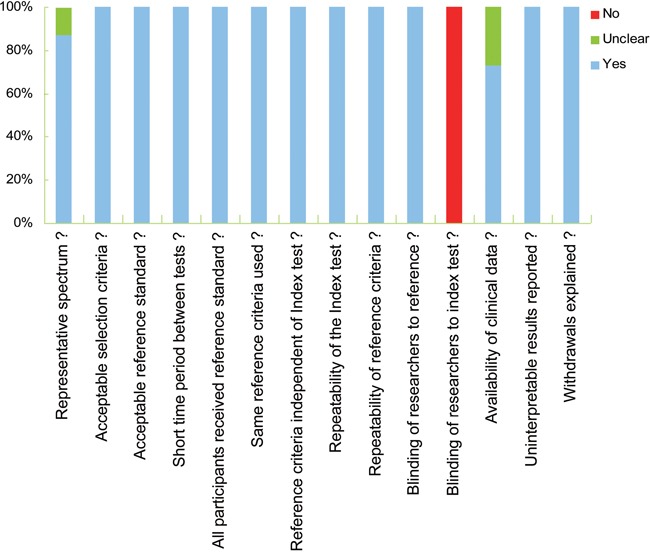
Study quality assessment using the QUADAS checklist

According to 14-item QUADAS checklist, all the 15 investigations achieved QUADAS scores equal or greater than 10, suggesting a relatively high quality of the included studies (Table 1 and Figure 2).

### Study heterogeneity

Study heterogeneity from threshold effects was reflected by Spearman correlation coefficient using Meta-Disc 1.4 software. As shown in Table 2, statistical analysis presented a Spearman correlation coefficient of 0.125, and P value of 0.633, indicating no obvious heterogeneity generated from threshold effect. In addition, the Cochran-Q test achieved a Q value of 51.46, and *P* value of 0.000, suggesting a likelihood of substantial heterogeneity generated by non-threshold effects. Additionally, heterogeneity from non-threshold effects seemed to exist in tissue-based assay as well (Table 2).

**Table 2:** Heterogeneity assessment of the pooled studies

Analysis	Spearman correlation Coefficient	Cochran's-Q test	*I*^2^ test (%)	Heterogeneity	
				Threshold effect	Non-threshold effect
Overall	0.125^a^	51.46^b^	68.9	No	Yes
	*P* = 0.633	*P* = 0.0000			
Plasma-based	0.400^a^	4.96^b^	39.6	No	No
	*P* = 0.600	*P* = 0.1745			
Tissue-based	0.126^a^	43.66^b^	72.5	No	Yes
	*P* = 0.681	*P* = 0.0000			

a Value of spearman correlation coefficient

b Q value

### Diagnostic performance

**Figure 3:**
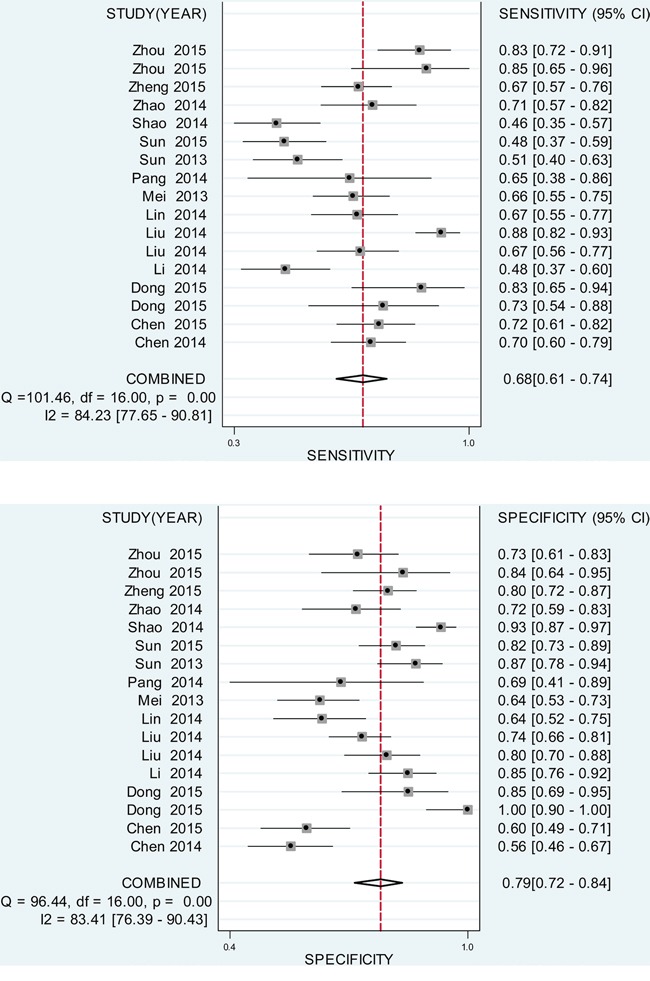
Forest plot of pooled sensitivity and specificity for the included studies A. pooled sensitivity. B. pooled specificity.

**Figure 4:**
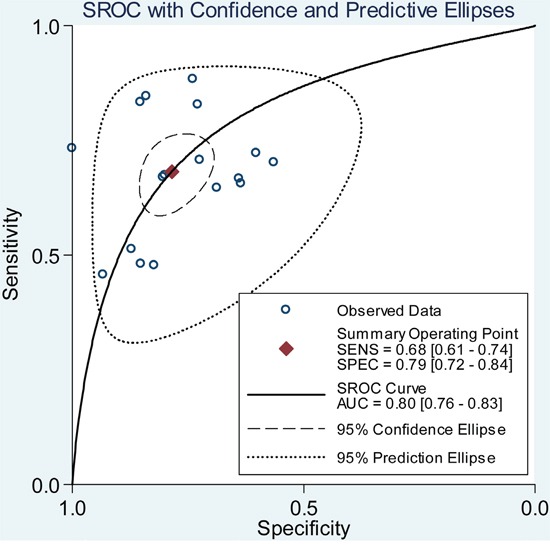
SROC curve for lncRNA expression profile in the diagnosis of GC

Since the existence of substantial heterogeneity among studies, a random-effect model was chosen for the generation of pooled indexes. The data showed that the combined sensitivity, specificity, PLR, NLR and DOR were 0.68 (95% CI: 0.61-0.74), 0.79 (95% CI: 0.72-0.84), 3.17 (95% CI: 2.46-4.10), 0.41 (95% CI: 0.33-0.49), and 7.83 (95% CI: 5.39-11.38), respectively. Forest plots of pooled sensitivity and specificity for lncRNAs are displayed in Figure 3. The SROC curve for the included studies is shown in Figure 4, in which lncRNA profiling yielded an AUC of 0.80, indicating a moderate accuracy for the diagnostic test.

### Subgroup analysis

Stratified analyses based on different specimen type demonstrated that plasma-based group achieved a higher accuracy than that tissue-based group: specificity 0.80 (95%CI: 0.75-0.85) versus 0.75 (95%CI: 0.73-0.78), PLR 3.33 (95%CI: 2.58-4.30) versus 2.7 (95%CI: 2.10-3.48), NLR 0.35 (95%CI: 0.21-0.60) versus 0.44 (95%CI: 0.36-0.54), DOR 9.47 (95%CI: 5.40-16.62) versus 6.69 (95%CI: 4.40-10.16), and AUC 0.84 versus 0.77, hinting that plasma may be a better matrix for the analysis of lncRNAs in conforming GC. However, the pooled sensitivity between such two groups was equally matched.

### Influence analysis and meta-regression

**Figure 5:**
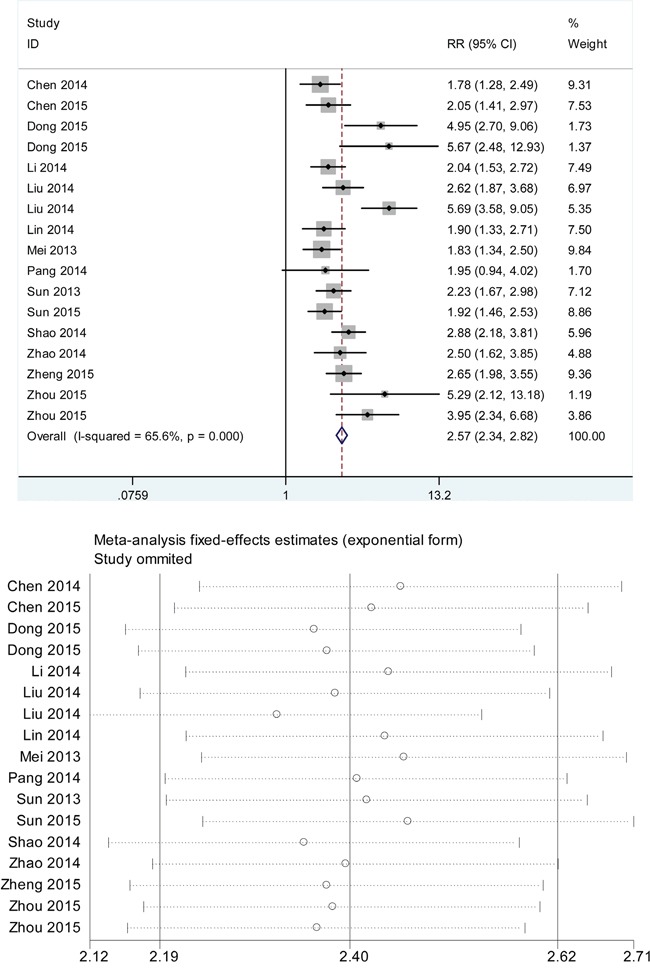
Influence analysis of the overall pooled study. **A. intermediate variable of RR; B. outlier detection analysis.** Influence analysis was conducted through STATA 12.0 software. RR: relative risk.

As indicated in Figure 5, the influence analysis identified no significant outlier studies, hinting that the outlier studies were not likely to be a source of heterogeneity. In addition, meta-regression revealed *P* values greater than 0.05 in all specified covariates, indicating that specimen type, control sources, sample size, reference gene, cut-off value and QUADAS score were unlikely to be the sources of heterogeneity (Table 3).

**Table 3:** Meta-regression for the potential source of heterogeneity

Study characteristic	*P*-value	RDOR	95% CI
Specimen type (plasma, serum or tissue)	0.0587	0.56	(0.31-1.02)
Control sources (healthy blood, normal tissue or adjacent non-tumor tissue)	0.9385	0.90	(0.05-15.68)
Sample size (GC<100 vs. GC≥100)	0.1047	2.27	(0.82-6.23)
Reference gene (GAPDH vs. β-actin)	0.5264	0.73	(0.25-2.13)
Cut-off value (value <100 vs. value ≥100)	0.1798	0.71	(0.42-1.2)
Study quality (QUADAS score≤10 or≥11)	0.1008	3.29	(0.76-14.14)

Abbreviations: CI: confidence interval; RDOR: relative diagnostic odds ratio; QUADAS: quality assessment for studies of diagnostic accuracy; GC: Gastric cancer; GAPDH: glyceraldehyde-phosphate dehydrogenase

### Publication bias

**Figure 6:**
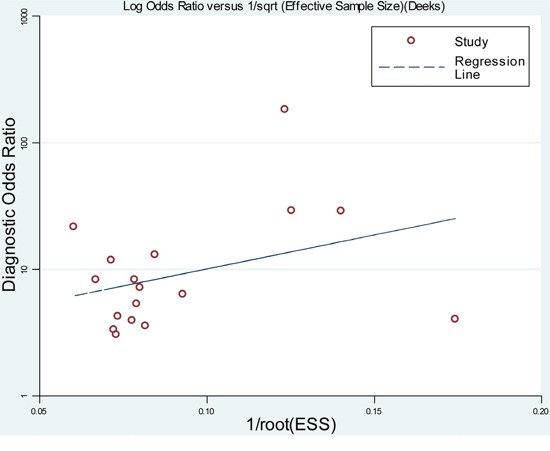
Funnel graph for the assessment of potential publication bias of the included studies

As shown in Figure 6, the slope coefficient did not reveal obvious evidences of asymmetry, with a *P* value of 0.548, suggesting that there was no potential publication bias among studies.

## DISCUSSION

Gastric cancer (GC) ranks the second leading cause of cancer deaths worldwide and early cancer detection remains a major challenge for GC research [1, 2]. Although the development of diagnostic methods and surgical techniques in recent years has remarkably improved the prognosis of GC patients, the 5-year survival rate for advanced GC still less than 30% [3]. The lack of diagnostic biomarkers accounts for the delay of early GC detection. Many non-invasive blood markers for GC detection are available thus far. For instance, PG, MG-7, CA19-9, CA72-4 and CEA are currently used in detecting GC [4–6]. Notwithstanding, these biomarkers are not ideal in confirming GC due to the relatively low diagnostic accuracies. On the other hand, endoscopic screening remains the most reliable diagnostic tool for GC detection, but it still yields the disadvantages of invasive status and relatively high costs [4]. It is therefore necessary to identify novel diagnostic biomarkers for GC screening.

It has become increasingly apparent that the versatile lncRNA reveals a diagnostic role in various kinds of cancers including GC [11–26]. In this study, we sought to evaluate the pooled diagnostic performance of lncRNAs for GC detection. The data manifested that the pooled sensitivity and specificity were 0.68 and 0.79, respectively, corresponding to an AUC up to 0.80. The diagnostic accuracy was estimated in a moderate level and it seems that the pooled sensitivity is not high enough for GC screening. On the other hand, the DOR (1.0 to infinity) is another indicator in mirroring discriminating power of a diagnostic test [27]. In this study, the DOR of lncRNAs was estimated to be 7.83, reflecting a moderate level of diagnostic accuracy. Additionally, a pooled PLR was estimated to be 3.17, suggesting that patients with GC had 3-fold higher chance of being lncRNAs positive (or significant changes) than non-GC cases. Meanwhile, a pooled NLR of 0.41 means that when the lncRNAs test is negative, 41% cases have the probability to be GC, which the value is not lower enough to rule out GC.

Data from the present meta-analysis suggest that lncRNAs expression profile yields a moderate diagnostic accuracy for GC. Although the relatively low pooled sensitivity and high NLR may not be powerful enough to diagnose GC, some points still support the utility of lncRNA(s) as an auxiliary biomarker for GC detection: First, most of the enrolled lncRNAs can distinguish healthy samples from early GC samples, suggesting a potential diagnostic value in GC. Second, lncRNAs were detectable in tumor tissues, peripheral blood even gastric juice from GC patients, and are not vulnerable to surrounding environment as well as other factors. Last, different lncRNAs should be investigated in panels, so as to select an optimum combination for potential clinical application. For example, a three-lncRNA signature (CUDR, LSINCT-5 and PTENP1) achieved a specificity of 100% and AUC of 0.92, with an overall diagnostic accuracy of 0.87 [21].

In the subgroup studies, a comparative analysis of lncRNA expression patterns in plasma and tissues manifested that plasma-based lncRNA profile harvested higher accuracy than tissue-based assay, suggesting that analysis using plasma may be better than tissue. The matrix differences have been confirmed in microRNAs by some meta-analysis studies [28, 29]. Similarly, one study documented that lncRNA test from sera samples yielded higher diagnostic accuracy than that from tissues, indicating that different specimen type may harvest different diagnostic accuracy [21]. However, only 4 individual studies were included for plasma-based lncRNA signature, which the accuracy might be comprised. Moreover, our stratified analyses failed to estimate the pooled accuracy for other matrix as serum or gastric juice due to a lack of sufficient data from publications. Thus, more studies are warranted to confirm this point.

In this study, we found significant heterogeneity appeared in the overall pooled study as well as the stratified analyses. Although all enrolled studies employed qRT-PCT method for the analysis of levels of lncRNA(s) expression, the reference gene differs among studies. It has been evidently reported that different reference gene may contribute to the accuracy in diagnosing GC [21]. Additionally, the samples size, specimen type as well as patient conditions for the tests were not relatively unified in studies. As a result, we conducted influence and univariate meta-regression analyses to trace the underlying heterogeneity sources. However, we found that the outlier study, study quality, specimen type, control type and sample size are not likely to be the sources of heterogeneity.

To conclude, our findings suggest that lncRNA expression profiles harbored a moderate accuracy in differentiation of GC patients and cancer-free individuals. LncRNA profiling reveals promising value in the management of GC. Nevertheless, several limitations were presented in our study. First of all, our analysis may have some population bias. Secondly, the control sources were complicated, for some are healthy blood or non-tumorous mucosa, and some are adjacent non-tumor tissues. More studies are therefore needed to highlight the value of lncRNAs as supplement test in diagnosing GC.

## MATERIALS AND METHODS

### Article search and inclusion criteria

This meta-analysis followed the standards of PRISMA (Preferred Reporting Items for Systematic Reviews and Meta-analysis) published in 2009 [30]. The online PubMed database and Foreign Medical Retrieval Service (FMRS) platforms were carefully searched for all suitable studies until September 30^th^ 2015. The search items were: “gastric cancer/carcinoma”, “long noncoding RNAs/lncRNAs”, “diagnosis/sensitivity/specificity/ROC/AUC”.

The enroll criteria were: studies (1) explored lncRNA(s) expression in GC subjects; (2) clearly defined study population and control sources; (3) explicitly defined sensitivity and specificity; and (4) published in English. The exclusion criteria were: studies (1) without complete data to tabulate 2 × 2 table; (2) had an unclear definition of the control group(s), or the controls sources were from tumors; and (3) reviews, meta-analyses, letters, commentaries, etc.

### Data extraction and study quality assessment

Data extracted from each study included the first author, publication date, country, control sources, sample size or types, detection method, lncRNA expression patterns and the diagnostic results. In case that study contains both training and validating tests, data from each test were extracted and deemed as an individual study. Any disagreements were resolved by discussion.

Studies quality was assessed following the checklist proposed by the evidence-based QUADAS tool [31]. According to the 14-items scoring criteria, each study was evaluated as “Yes (high concern)”, “No (low concern)” or “Unclear (unclear concern/risk)”, corresponding to a score of “1”, “0” and “0”, respectively.

### Statistical analysis

The STATA 12.0 (Stata Corporation, College Station, TX, USA) and Meta-Disc 1.4 (XI Cochrane Colloquium, Barcelona, Spain) software were used for the statistical analyses. The pooled sensitivity, specificity, diagnostic odds ratio (DOR), positive likelihood ratio (PLR), negative likelihood ratio (NLR) were generated using a bivariate analysis. Heterogeneity from threshold and non-threshold effects was separately assessed by Spearman correlation coefficient, Cochran-Q and Inconsistency index (I^2^) tests. When a significant heterogeneity exists among studies (P < 0.05 for Cochran-Q test or I^2^ > 50%), a random-effect model will be chosen for the generation of pooled indexes [32]. The potential publication bias was analyzed by Deeks' funnel plot asymmetry test and the significant level was set in P < 0.01. Influence and univariate meta-regression analyses were performed to trace the potential heterogeneity sources. The covariates of meta-regression involved sample size (<100 or ≥100) [33], specimen type (plasma, serum, tissue or other), control sources (healthy blood, normal tissue or adjacent non-tumor tissue), reference gene (GAPDH or β-actin), cut-off value (<10 or ≥10), and study quality (QUADAS socre≤10 or≥11).
